# Advancing Antimicrobial Textiles: A Comprehensive Study on Combating ESKAPE Pathogens and Ensuring User Safety

**DOI:** 10.3390/ma17020383

**Published:** 2024-01-12

**Authors:** Kinga Vojnits, Majid Mohseni, Mazeyar Parvinzadeh Gashti, Anupama Vijaya Nadaraja, Ramin Karimianghadim, Ben Crowther, Brad Field, Kevin Golovin, Sepideh Pakpour

**Affiliations:** 1 School of Engineering, University of British Columbia, Kelowna, BC V6T 1Z2, Canada; kinga.vojnits@ubc.ca (K.V.); ramin.karimianghadim@ubc.ca (R.K.); nebben13@hotmail.com (B.C.); 2 Research and Development Laboratory, PRE Labs, Inc., Kelowna, BC V1X 7Y5, Canada; majid.mohseni1@ucalgary.ca; 3 GTI Chemical Solutions, Inc., Wellford, SC 29385, USA; mparvinzadeh@gmail.com; 4 InsectaPel, LLC, Wellford, SC 29585, USA; 5 Mechanical and Industrial Engineering, University of Toronto, Toronto, ON M5S 3G8, Canada; anu.pillai@utoronto.ca (A.V.N.); kevin.golovin@utoronto.ca (K.G.); 6 PRE Labs, Inc., Kelowna, BC V1X 7Y5, Canada; bfield@prelabscanada.com

**Keywords:** antimicrobial textiles, cytotoxicity, human dermal fibroblasts, multivariate analysis

## Abstract

Antibiotic-resistant bacteria, ESKAPE pathogens, present a significant and alarming threat to public health and healthcare systems. This study addresses the urgent need to combat antimicrobial resistance by exploring alternative ways to reduce the health and cost implications of infections caused by these pathogens. To disrupt their transmission, integrating antimicrobial textiles into personal protective equipment (PPE) is an encouraging avenue. Nevertheless, ensuring the effectiveness and safety of these textiles remains a persistent challenge. To achieve this, we conduct a comprehensive study that systematically compares the effectiveness and potential toxicity of five commonly used antimicrobial agents. To guide decision making, a MULTIMOORA method is employed to select and rank the optimal antimicrobial textile finishes. Through this approach, we determine that silver nitrate is the most suitable choice, while a methoxy-terminated quaternary ammonium compound is deemed less favorable in meeting the desired criteria. The findings of this study offer valuable insights and guidelines for the development of antimicrobial textiles that effectively address the requirements of effectiveness, safety, and durability. Implementing these research outcomes within the textile industry can significantly enhance protection against microbial infections, contribute to the improvement of public health, and mitigate the spread of infectious diseases.

## 1. Introduction

Antibiotic-resistant bacteria, known as ESKAPE (*Enterococcus faecium*, *Staphylococcus aureus*, *Klebsiella pneumoniae*, *Acinetobacter baumannii*, *Pseudomonas aeruginosa*, *Enterobacter* spp., and *Escherichia coli*) pathogens, pose a significant challenge to healthcare and public health [1,2,3,4]. Reports estimate over 2 million antimicrobial-resistant infections occur annually in the United States, resulting in healthcare costs of approximately USD 4.7 billion [5]. Extensive research has been conducted to understand the mechanisms of resistance exhibited by ESKAPE pathogens and to explore alternative approaches, in combination with antibiotics, to reducing the health and cost implications of infectious diseases caused by these pathogens. These alternative approaches include bacteriophage therapy [6], usage of antimicrobial peptides [7], photodynamic therapy [8], usage of antibacterial antibodies [9,10], as well as the application of nanoparticles [5,10,11,12] and phytochemicals [13]. Advanced porous materials, such as mesoporous silica nanoparticles, porous silicon nanoparticles, metal–organic frameworks, covalent–organic frameworks, hydrogen-bonded organic frameworks, and porous carbon materials, have also emerged as promising alternatives due to their low cost of production, high biocompatibility, adjustable porous structure, large surface area, and easy surface functionalization [14]. These materials have shown great potential as nanotools for antimicrobial treatment, although only a few examples have been currently described in the literature [15,16,17]. Additionally, the current progress towards the design of these advanced porous materials for antimicrobial treatment is mainly focused on in vitro antimicrobial evaluation, with only a few in vivo examples reported so far. Due to their utility in various industrial applications, nanoparticles, materials with a three-dimensional basic unit that falls within a range between 1 and 100 nm, offer promising potential as antimicrobial agents against bacterial and viral infections [18,19]. The physicochemical properties of nanoparticles underlying their antimicrobial activity include their size, charge, surface morphology, crystal structure, and zeta potential [20]. Their small size is their main property that confers excellent antimicrobial action and effective combating of intracellular bacteria, as it facilitates the penetration of nanoparticles through bacterial cell walls into the bacteria. Although the exact mechanisms underlying the antimicrobial activity of nanoparticles have not been conclusively elucidated, some evidence has suggested that their antimicrobial activity solely depends on the production of reactive oxygen species or the development of oxidative stress that leads to apoptosis [21]. Moreover, research is still underway on whether the toxicity of nanoparticles originates from ions released from nanoparticles or the nanoparticles themselves. Hence, the detailed antibacterial mechanisms of nanoparticles are worth considering in future research.

Simultaneously, there has been a significant focus on the development of rapid-contact-killing antimicrobial surfaces to disrupt the transmission of these dangerous pathogens [22,23,24]. This includes the integration of antimicrobial textiles into wearable personal protective equipment (PPE), as textiles can act as reservoirs for microorganisms during usage and storage, facilitating the spread of infections [25,26]. Notably, the global antimicrobial textiles market is expected to grow from USD 10.7 billion to USD 14.7 billion by 2026, with a compound annual growth rate (CAGR) of 6.5% from 2021 to 2026 [27].

The antimicrobial activity of functional textiles depends on multiple factors including degree of adherence and survival of pathogens on textile substrates, the nature of antimicrobial agents, and the durability of antimicrobial agents for different textile applications [25,28]. The major classes of synthetic and natural antimicrobial agents for textiles include triclosan, metals and their salts, organometallics, phenols, quaternary ammonium compounds (QACs), organosilicons, and essential oils [28,29,30]. Among these, the application of silver nanoparticles and zinc-based salt solutions on textiles have shown a broad-spectrum effect on different microorganisms including Gram-positive and Gram-negative bacteria, fungi, viruses, yeast, algae [31], and most recently SARS-CoV-2 [32]. However, it is important to note that both silver and zinc salts have been found to be toxic [33]. A recent analysis assessing the environmental risks associated with these substances in textiles recommends that the potential release of these compounds during washing should be determined prior to marketing and use [34]. QACs are also widely used biocides that possess antimicrobial efficacy against a broad range of microorganisms with a primary mode of action being structure/function disruption against cell membranes [35]. However, the antimicrobial range of QACs has been shown to be less than that of oxidizing disinfectants [36,37].

As an alternative approach, researchers have also been exploring the use of natural antibacterial agents, such as plant-based products, e.g., aloe vera, tea tree oil, and eucalyptus oil, which are known for their antioxidant properties [38]. They have demonstrated high antimicrobial performance against several pathogenic bacteria and yeasts such as *Bacillus subtilis*, *Escherichia coli*, *Pseudomonas aeruginosa*, *Staphylococcus aureus*, and *Candida albicans* [39]. However, durability and resistance to washing remains the weak point for antimicrobial finishes based on natural agents. This weakness results from their volatility, sensitivity to light and oxygen, and from their difficulty in forming bonds with textile materials [40,41]. In addition, there is currently a gap in the research regarding the efficacy of textiles functionalized with different antimicrobial agents. To date, no studies have directly compared the effectiveness of various antimicrobial agents when integrated into textile materials. This gap in knowledge hinders our understanding of which antimicrobial agents are most effective in preventing the spread of infections caused by ESKAPE pathogens through textiles, such as those used in PPE. In addition, while antimicrobial properties are important for preventing the spread of pathogens, it is equally important to ensure that the textiles used in PPE are safe and gentle on the skin. This entails conducting thorough assessments to determine the potential adverse effects and irritability of these textiles when in contact with human skin. Such evaluations should focus on parameters like cytotoxicity, skin irritation, and allergic reactions to ensure that the antimicrobial textiles are not harmful or discomforting to the wearer.

In order to bridge these existing gaps, our research focuses on the development of antimicrobial textiles that effectively combat pathogens while simultaneously prioritizing user comfort, performance, and cost effectiveness. To achieve this, we conducted a comprehensive study that involved evaluating various textiles that were functionalized with different antimicrobial agents targeting ESKAPE pathogens. In addition to assessing their efficacy, we also examined the antimicrobial durability and biocompatibility of these textiles. Furthermore, we employed a mathematical decision aid tool to facilitate the selection of the optimal antimicrobial agent. This tool considered multiple criteria such as effectiveness, safety, heat sensitivity, odor, and price. By considering these essential factors, our research aims to contribute to the advancement of antimicrobial textiles that not only meet stringent standards but also offer enhanced protection in healthcare and other relevant settings.

## 2. Materials and Methods

### 2.1. Materials

A 500-denier 91:9% nylon:spandex plain-weave textile with a weight of 100 g/m^2^ was supplied by Stedfast Inc. (Granby, QC, Canada). Zinc chloride, anhydrous (ZnCl_2_, 97%, Alphachem, Mississauga, ON, Canada), silver nitrate (AgNO_3_, 99.5%, Thermo Fisher Scientific, Waltham, MA, USA), tea tree oil (Sigma-Aldrich, Burlington, MA, USA), trimethoxysilylpropyl octadecyldimethyl ammonium chloride (HM4072, 72%, Gelest, Morrisville, PA, USA), and (trihydroxysilyl) propyldimethyl octadecyl ammonium chloride (HM4005, 84%, Gelest, Morrisville, PA, USA) were purchased and used as antimicrobial agents.

### 2.2. Preparation of Antimicrobial Textiles

**Figure 1:**
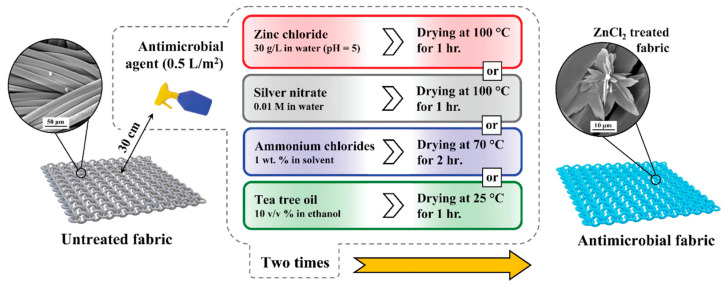
Schematic presentation of the general procedure for antimicrobial fabric preparation.

Circular textile swatches with a diameter of 4 cm were prepared, washed in a 1 wt.% commercial detergent bath for 30 min, and rinsed twice with warm water at 50 °C to eliminate any contaminants. Various groups of antimicrobial agents, including metal-based, QACs, and plant-based options, were applied to the washed fabric swatches using a scalable and controllable spraying method. The zinc chloride spray was prepared by dissolving ZnCl_2_ in water (30 g/L) and adjusting the pH to 5 using acetic acid. For the silver-based coating, a 0.01 M silver nitrate (AgNO_3_) solution was utilized. The QACs HM4072 and HM4005 were diluted to 1 wt.% in methanol and water, respectively, following the manufacturer’s recommendations. Tea tree oil was dissolved in ethanol (10% *v*/*v*) before application. These antimicrobial solutions were sprayed onto the fabric swatches using a power handy sprayer with a uniform spray coverage of 0.5 L/m^2^. The sprayer nozzle was kept at a distance of 30 cm from the target, and the coating and drying processes were repeated twice to ensure complete coverage of the textiles (Figure 1).

### 2.3. Antibacterial Assessment on Coated Textiles

The antimicrobial activity of the textiles coated with zinc chloride, silver nitrate, QACs (HM4072 and HM4005), or tea tree oil was assessed against three multidrug-resistant ESKAPE pathogens, *Escherichia coli* (*E. coli*, ATCC 11229), *Klebsiella pneumonia* (*K. pneumonia*, ATCC 4352)*,* and Gram-positive *Staphylococcus aureus* (*MRSA*, ATCC 6538). The test organisms were purchased from American Type Culture Collection (ATCC). *E. coli* and *K. pneumonia* were cultured in their defined growth medium, Nutrient Broth (Thermo Fisher Scientific) on nutrient agar plates, and *MRSA* was cultured in Tryptic Soy Broth (Thermo Fisher Scientific) on tryptic soy agar plates. The fresh bacterial culture was prepared by growing it in Nutrient Broth or Tryptic Soy Broth medium at 37 °C overnight in an incubator shaker at 150 rpm. The bacterial cell density was adjusted to 1.0 × 10^5^ cells/mL based on the optical density reading at 600 nm (OD_600_).

A modified AATCC-100 standard colony-counting method was used to conduct antimicrobial assays. Briefly, textile swatches were added to the growth medium, inoculated with individually tested organisms with the same initial inoculum concentration (1.0 × 10^5^ cells/mL), and incubated for 24 h at 37 °C in an incubator shaker at 150 rpm. Flasks containing only growth medium with samples were used as controls. After 24 h incubation, serial dilutions of the liquid were made in sterilized water. Dilutions of 10^−2^, 10^−4^, and 10^−6^ were subjected to colony-counting tests. For this, 100 μL was spread onto the agar plates, nutrient agar for *E. coli* and *K. pneumonia* and tryptic agar for *MRSA*, and then incubated at 37 °C for 24 h. After incubation, bacterial colonies were counted and recorded as colony-forming unit/mL (CFU/mL). The percent reduction in the number of colonies in the treated samples as compared to the untreated samples provides the antibacterial activity of the treated textile.

Antimicrobial activity or % bacteriostatic reduction (BR) was calculated according to Equation (1):

(1) $$100\left(B-A\right)/B=BR$$

where *BR* = % bacteriostatic reduction, *A* = the number of bacteria recovered from the inoculated treated test specimen swatches incubated over the 24 h contact period, and *B* = the number of bacteria recovered from the inoculated untreated test specimen swatches incubated over the 24 h contact period.

### 2.4. Textiles Leachate Preparation

To prepare leachate solutions, sterilized antimicrobial textile swatches were dipped in 3 mL of Iscove’s Modified Dulbecco’s Medium (IMDM, Thermo Fisher Scientific) supplemented with 10% fetal bovine serum (FBS, Thermo Fisher Scientific) and incubated at 37 °C in 5% CO_2_ for 2 h, 6 h, and 24 h. The leachate solution was collected in universal tubes for use in the cytotoxicity studies.

### 2.5. Cell Culture and Cytotoxicity Assessment

Normal human adult dermal fibroblast cells (HDFs; line CCD-1064Sk—ATCC CRL-2076) were used as a model of dermis cells. The cells were maintained in IMDM supplemented with 10% FBS at 37 °C and 5% CO_2_. The HDFs were sub-cultured every 6 days.

**Figure 2:**
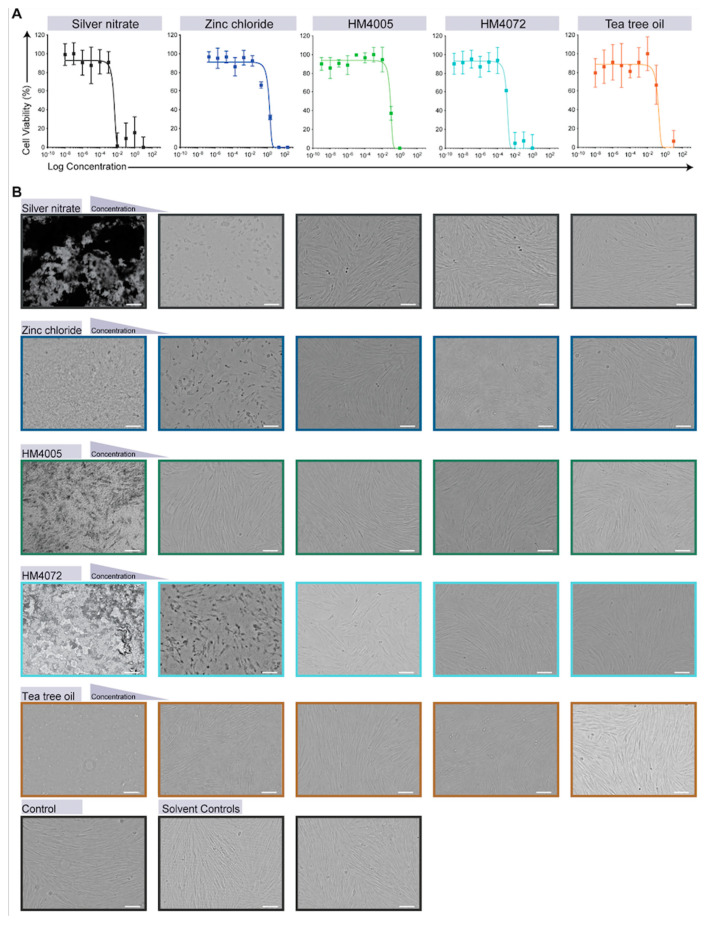
(**A**) Dose-response curves of human dermal fibroblast (HDF) cell viability measured by resazurin assay of zinc chloride, silver nitrate, HM4005, HM4007, and tea tree oil solution after 1 day of exposure. Data show mean ± SE. (**B**) Microscopic bright-field images of control HDF and HDF cultures after 1 day of exposure to different concentrations of zinc chloride, silver nitrate, HM4005, HM4007, and tea tree oil solutions. Scale bars = 100 μm.

For toxicity assays, the HDFs were washed with phosphate-buffered saline (PBS) and harvested using 0.25% trypsin-EDTA solution (Thermo Fisher Scientific). After trypsinization, counting, and appropriate dilutions, the cells were seeded at a density of 5.0 × 10^3^ cells/well in a 96-well tissue culture-treated polystyrene plate. After 24 h, when the HDFs had attached to the surface of the plate, the cells were treated with the antimicrobial agents alone in 10-fold serial dilutions to determine the IC_50_ concentrations (Figure 2A, Table 1) and assess any morphological alterations (Figure 2B).

For textile leachate cytotoxicity assessment, HDFs were treated with the leachate medium solution from the test textiles and incubated for 24 h at 37 °C and 5% CO_2_. Cells without extracts were used as negative control wells. For positive controls, 30% DMSO, as a known cytotoxic compound at this concentration for HDFs, was used. The cytotoxicity of the antimicrobial agents or the test textiles was evaluated after 24 h of exposure using the resazurin assay, measuring the fluorescence signal at 544 nm excitation and 590 nm emission, and MTT cell viability assays, measuring absorbance at 570 nm, using a microplate reader (Tecan Group LTD, Mannedorf, Switzerland). The percentage of cell viability was calculated according to the following formula: 100 × (Mean treated sample values/Mean control sample values).

### 2.6. Statistical Analysis

A minimum of three biological replicates were used in each experiment. Statistical analyses were carried out using GraphPad Prism version 9.3.1 (Dotmatics, Boston, MA, USA). All numerical data are expressed as mean values ± SE. Data normality was assessed using the Shapiro–Wilk test (Shapiro–Wilk: *p* > 0.05). Comparisons between control/untreated and treated samples were performed by one-way analysis of variance (ANOVA) followed by Bonferroni correction. Statistical significance was considered at *p* < 0.05, where * *p* < 0.05, ** *p* < 0.01, *** *p* < 0.0002, and **** *p* < 0.0001.

### 2.7. Multicriteria Decision-Making (MCDM) Analysis

Choosing the best candidate when considering diverse and conflicting factors is a typical multicriteria decision-making (MCDM) problem. MCDM methods assist decision-makers when there is no dominance of one option over others and when there is a trade-off in selecting feasible alternatives. There have been many MCDM methods developed over the years, including the MOORA method, which is one of the well-developed MCDM techniques. Exploiting its simplicity, short computational time, high stability, and minimal amount of mathematical calculations, the MOORA method has resolved the criteria-weighting issues of other MCDM methods, such as TOPSIS, PROMETHEE, ELECTRE, and AHP [42,43]. The MOORA method was first introduced in 2006 (ratio system and the reference point method). It was then further developed into a full multiplicative form and extended into the MULTIMOORA [44,45]. The MULTIMOORA method is achieved by adding the full multiplicative form and applying the dominance theory to integrate the results of these three subordinate techniques to obtain the final rankings. Regarding the performance of the proposed MOORA methods, the MOORA ratio method performs well when there are no “dependent” criteria in the decision matrix. However, the fully multiplicative form has a desired performance in the case of dependent criteria. When the decision-maker seeks a solution that does not have a bad performance in any of the criteria, the reference point MOORA could be an appropriate method. Integrating the outcome of the three MOORA versions using the dominance theory, the MULTIMOORA method benefits from all three methods. MULTIMOORA has been applied in a wide variety of areas such as economics [46], civil services [47], and the healthcare system [48,49].

The top-ranked antimicrobial agents were obtained using the MCDM approach. Starting with a decision matrix X with *m* alternatives and *n* criteria, in which $x_{ij}$ is the response of alternative *j* on criterion *i*, the MULTIMOORA method consists of three parts: MOORA ratio system (MOORA_ratio), MOORA reference point approach (MOORA_refpoint), and MOORA full multiplicative form (MOORA_fmf), which are described as follows.

I. The MOORA ratio system applies the vector normalization according to Equation (2):
  (2) $$x_{ij}^{*}=\frac{x_{ij}}{\sqrt{\sum_{j=1}^{m}x_{ij}^{2}}},\;\;\;\;\;\;\;\;\;\;\;\;\;\;\forall \;i,j$$

Afterward, the index of each criterion is calculated using Equation (3):

(3) $$y_{j}^{*}=\overset{t}{\underset{i=1}{\sum }}x_{ij}^{*}-\overset{n}{\underset{i=t+1}{\sum }}x_{ij}^{*},\;\;\;\;\;\;\;\;\;\;\;\;\forall \;i$$

The beneficial criteria are added to the index and the non-beneficial ones are subtracted. Finally, the rankings are obtained by the index ratio, in which a higher index yields a higher rank.

II. The MOORA reference point approach is based on the MOORA ratio system. Following the values obtained from Equation (3), the coordinates for criteria are calculated based on Equations (4) and (5) for beneficial and non-beneficial criteria, respectively. Then, based on the deviation for the reference point and the min–max metric, the final rankings are obtained using Equation (6):
  (4) $$r_{i}=\underset{j}{\max}x_{ij}^{*}$$
  (5) $$r_{i}=\underset{j}{\min}x_{ij}^{*}$$
  (6) $$\underset{j}{\min}\left\{\underset{i}{\max}\left|r_{i}-x_{ij}^{*}\;\right|\right\}$$
III. In the MOORA full multiplicative form method, the overall utilities of the alternatives are obtained using Equation (7), which includes the maximization as well as the minimization of beneficial and non-beneficial objectives, respectively:

(7) $$U_{j}^{\prime }=\frac{A_{j}}{B_{j}}\;,\;j=1,\;2,\;\ldots ,\;m$$

with $A_{j}=\overset{i}{\underset{k=1}{\prod }}x_{kj}$ (*i* = number of criteria to be maximized) and $B_{j}=\overset{n}{\underset{k=i+1}{\prod }}x_{kj}$ (*n*−*i* = number of criteria to be minimized).

Finally, using the previously developed dominance theory [45], the MULTIMOORA method summarizes the results of the three methods, which leads to the most robust approach of multiple-objective optimization [50]. Moreover, in this study, we included weighting based on experts’ opinions for the final ranking. Six experts from different domains, three representing health scientists and engineers, and three being our industry partners, assigned numerical weights to each criterion, with the weights summing up to one. By considering the contribution of each expert, the weighted average of their inputs was calculated as the final output.

## 3. Results

**Figure 3:**
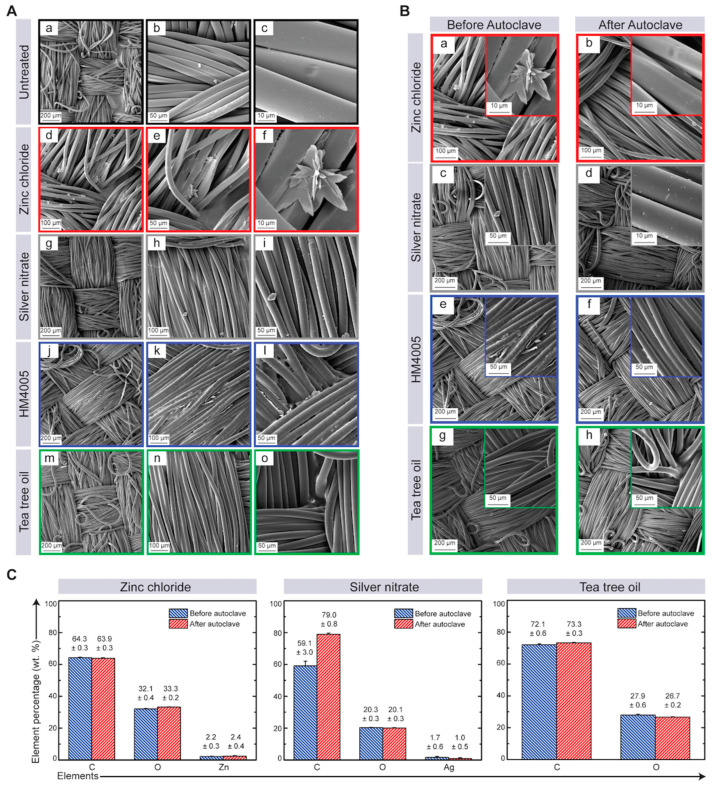
Evaluation of the coated layer and mechanical characterization. (**A**) SEM images of (**a**–**c**) untreated fibers (black squares), (**d**–**f**) textiles treated with zinc chloride (red squares), (**g**–**i**) textiles treated with silver nitrate (grey squares), (**j**–**l**) HM4005-treated textiles (blue squares), and (**m**–**o**) textiles treated with tea tree oil solution (green squares) at various magnifications. (**B**) SEM images of textiles treated with zinc chloride (**a**) before and (**b**) after autoclave; silver nitrate (**c**) before and (**d**) after autoclave; HM4005 (**e**) before and (**f**) after autoclave, and tea tree oil (**g**) before and (**h**) after autoclave. Larger magnifications are presented in insets. (**C**) Elemental composition of textiles sprayed with zinc chloride, silver nitrate, and tea tree oil before and after autoclave from EDS spectrum. In the elemental analysis, the reported values represent the mean and standard deviation of at least three measurements.

The surface morphology and elemental composition of the untreated and antimicrobial-coated textiles were analyzed using scanning electron microscopy (SEM, Tescan Mira 3 XMU, Tescan, Brno, Czech republic) equipped with energy-dispersive X-ray spectroscopy (EDS). The untreated textiles exhibited a relatively smooth surface without any particle deposition (Figure 3A). The zinc chloride-coated textiles showed the presence of star-shaped particles (Figure 3B), and the zinc content was recorded as 2.3 wt.% using EDS (Figure 3C). The surface of the silver nitrate-coated textiles showed deposition of some particles, and a silver content of 2.1 wt.%, as measured using EDS. The HM4005-coated textiles showed a uniform coating with silicon and chlorine contents of 3.8 wt.% and 3.9 wt.%, respectively (Figure 3C). The tea tree oil-coated textiles showed no changes in the surface morphology of fibers (Figure 3A–C). Moreover, heat sterilization and autoclaving did not affect the surface morphology or elemental composition of the antimicrobial-coated textiles (Figure 3B,C).

### 3.1. Assessment of Efficacy of Antimicrobial-Functionalized Textiles in Controlled Environments

**Figure 4:**
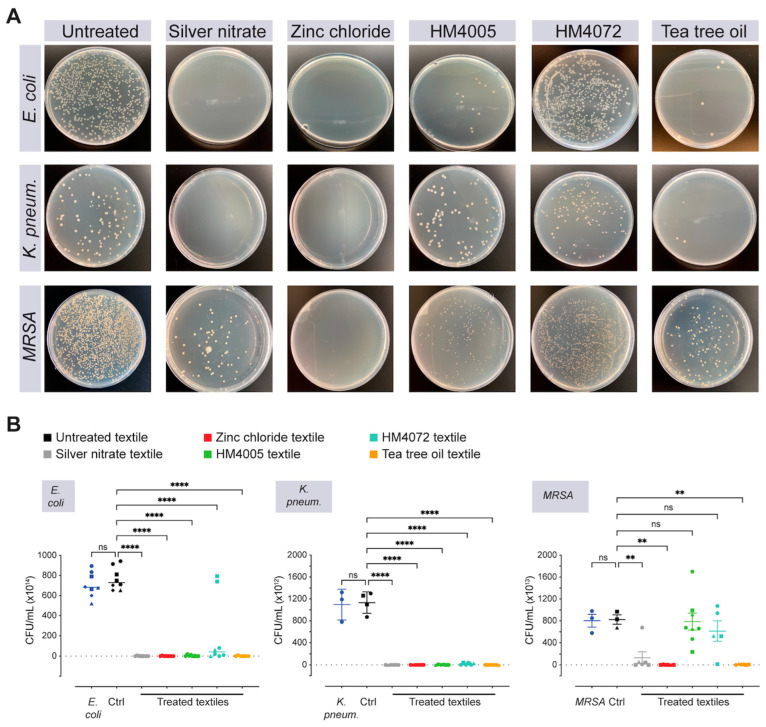

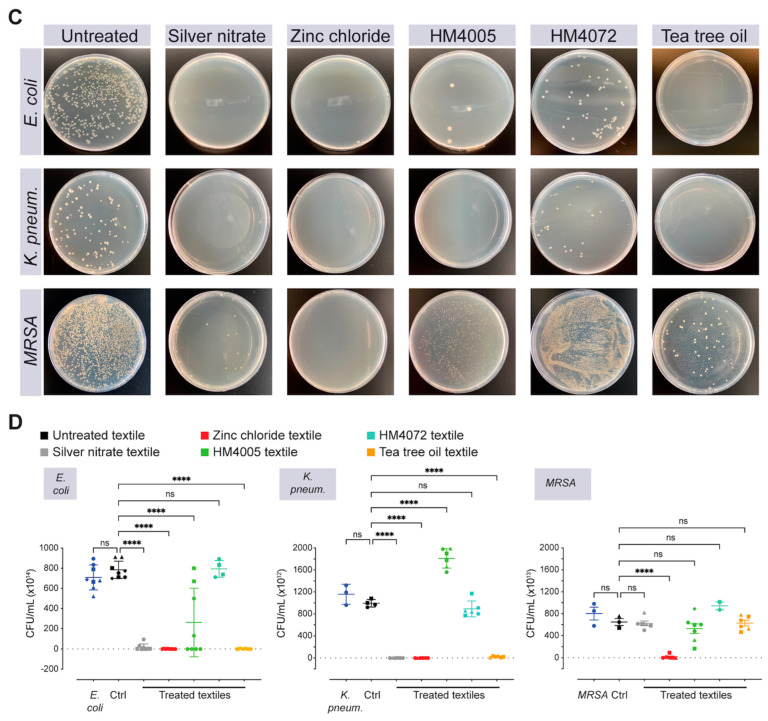
Antimicrobial activity of the fabric layer (500-denier Cordura) spray-coated with selected antimicrobial agents, with comparison to untreated textile with the AATCC 100 test. (**A**) Optical images with Gram-negative bacteria *Escherichia coli* and *Klebsiella pneumoniae*, and Gram-positive *Staphylococcus aureus (MRSA)*. (**B**) Whisker plots show colony-forming units (CFUs) of the different tested bacteria species, *Escherichia coli* (*E. coli*), *Klebsiella pneumoniae* (*K. pneum.*), and *Staphylococcus aureus (MRSA)*. (**C**,**D**) Effect of sterilization on the antimicrobial activity. Optical images and whisker plots show the assessment of colony formation of the tested bacteria species. Colony formation was analyzed and counted using semi-automated image analysis. Data show mean ± SE, ** *p* < 0.01, and **** *p* < 0.0001 compared to untreated textile. Different symbol shapes represent different textile swatches.

Evaluating the combined effect of direct contact and leachates, the antimicrobial assessment of textiles coated with silver nitrate, zinc chloride, tea tree oil, and QACs (HM4005 and HM4072) against *E. coli*, *K. pneumonia*, and Gram-positive *MRSA* revealed most of the tested agents had antimicrobial activity against both Gram-negative and Gram-positive bacteria species (Figure 4A,B). Silver nitrate, zinc chloride, and tea tree oil had a 99% bacteriostatic reduction rate (BR), indicating excellent antimicrobial activity (Figure 4A,B, Table 2), whereas the QACs (HM4005 and HM4072) were effective only against the tested Gram-negative bacterial species (>80% BR) but not against the Gram-positive *MRSA* (0% and 25% BR by HM4005 and HM4072, respectively; Table 2).

### 3.2. Impact of Heat Sterilization on the Durability of Antimicrobial-Coated Textiles

The durability of antimicrobial treatments is crucial for providing long-term protection. However, heat sterilization can potentially affect the performance of antimicrobial agents, as heat can degrade molecules and textiles, especially in healthcare settings that undergo frequent sterilization procedures. To investigate this, a durability study was conducted on antimicrobial-coated textiles after autoclaving at 121 °C and 15 psi for 15 min. The results demonstrate that sterilization can have a significant impact on antimicrobial activity, with quaternary ammonium compounds (QACs) showing the least activity post-sterilization (Figure 4C,D; Table 2). However, silver nitrate and tea tree oil maintained their antimicrobial activity against Gram-negative bacteria (except *MRSA*) even after sterilization. Among the tested agents, textiles coated with zinc chloride exhibited the highest activity against all tested bacteria species, indicating it to be the most durable antimicrobial agent (Figure 4C,D).

### 3.3. Assessment of the Cytotoxicity of Leachates from Antimicrobial-Functionalized Textiles on Human Dermal Fibroblasts

**Figure 5:**
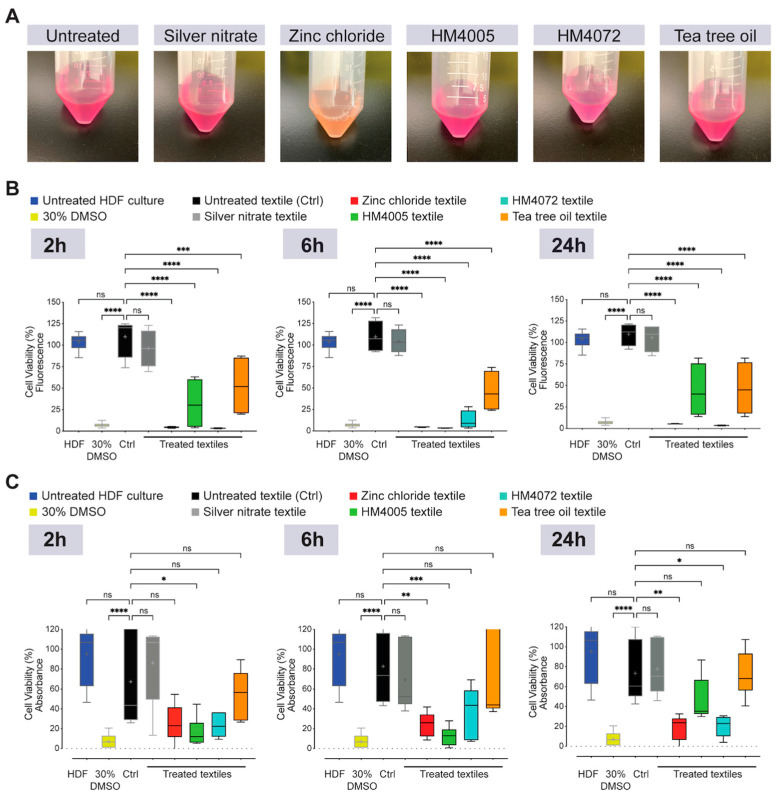
Biocompatible assessment of tested antimicrobial agents. (**A**) Images of leachates prepared from textiles coated with the different antimicrobial agents. Human dermal fibroblast (HDF) cell viability measurements using resazurin assay (**B**) and MTT assay (**C**). Whisker plots show HDF cell viability after 1 day of exposure with the suspension from antimicrobial-agent-impregnated textiles from different fabric–media incubation periods. Data show mean ± SE, * *p* < 0.05, ** *p* < 0.01, *** *p* < 0.0002, and **** *p* < 0.0001, compared to untreated HDF cells.

To evaluate the impact of antimicrobials on human skin, cytotoxicity assays (resazurin and MTT) were performed on human dermal fibroblasts (HDFs) using leachate extracted from antimicrobial-coated textiles. An optical evaluation showed color changes and visible particles in the leachate media prepared from zinc chloride-, HM4005-, and HM4072-coated textiles (Figure 5A). No antimicrobial agent leaching was observed from the silver nitrate- or tea tree oil-coated textiles during the 24 h leachate preparation (Figure 5A). Moreover, the cytotoxicity assay data demonstrated that HDFs maintained 100% cell viability even after exposure to leachate from silver nitrate-coated fabric for 24 h (Figure 5B,C). Tea tree oil did not significantly affect the cell viability as more than 50% of cells remained viable at all tested timepoints. In contrast, leachates from zinc chloride- and HM4072-coated textiles exhibited significant cytotoxicity (Figure 5B,C). HM4005 showed lower toxicity compared to zinc chloride, resulting in a 60% reduction in HDF cell viability (Figure 5B,C).

### 3.4. Optimal Antimicrobial Textile Selection Using Multicriteria Decision-Making Methods

Multicriteria decision-making methods were utilized in this research to address the presence of conflicting multiple criteria, facilitating a systematic evaluation and selection of optimal antimicrobial textile options that strike a balance between different objectives. Specifically, in the material selection problem, a decision matrix was created to represent the alternatives and their corresponding criterion values (Table 3).

**Figure 6:**
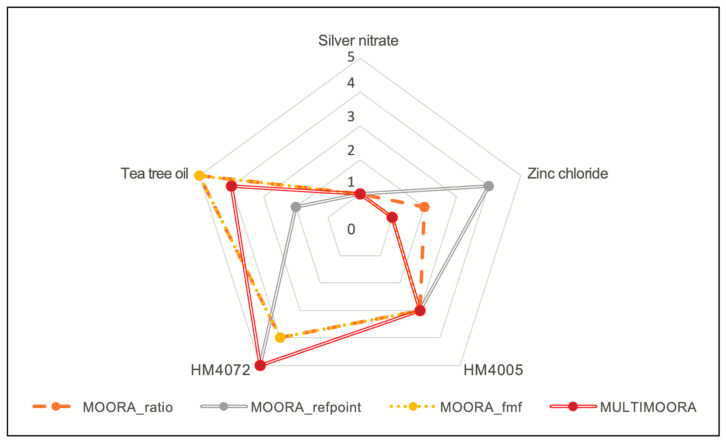
Assessment model for choosing the optimal coating material based on multivariate criteria. The radar chart reflects the comparison of the MCDM results for the material selection problem. The chart has five layers, with the outermost layer corresponding to the highest rank (worst alternative) and the innermost one representing the lowest rank (best alternative) for the existing options.

Experts from different domains assigned numerical weights to each criterion, with the weights summing up to one. By considering the contribution of each expert, the weighted average of their inputs was calculated as the final output (Table 4). Using the MOORA_ratio, MOORA_refpoint, MOORA_fmf, and MULTIMOORA methods on the decision matrix, the results are summarized in Figure 6 using a radar chart, with silver nitrate as the top-ranked option, followed by zinc chloride and HM4005. Tea tree oil and HM4072 were found to be the least favorable alternatives, except for when using the MOORA_refpoint method, which ranked HM4072 as the second-best option. Thus, based on the results from these methods, silver nitrate emerged as the ideal choice, while HM4072 was deemed the least suitable for the material selection problem.

## 4. Discussion

To address the growing challenges posed by microbial infections, multidrug-resistant bacteria, and the emergence of new viruses, the adoption of appropriate antibacterial textiles and protective garments has become crucial in hospitals and public settings [51,52,53]. Personal protective equipment (PPE) enhanced with antimicrobial agents has shown potential in safeguarding against a wide range of pathogenic microorganisms. However, the effectiveness and level of protection depend on various factors, primarily the use of suitable antimicrobial agents on textiles [28]. Despite the undeniable potential of employing these agents for antimicrobial textile finishes to combat ESKAPE pathogens and nosocomial infections, their application and integration within the textile industry have been impeded by several challenges.

One major obstacle is the requirement for broad-spectrum activity. In our research, we have demonstrated the effectiveness of various antimicrobial agents with wide-ranging applications. Silver nitrate, zinc chloride, and tea tree oil have been identified as strong active antimicrobial agents. Consistent with the existing literature [54,55,56,57,58], our research findings highlight the outstanding antimicrobial activity of silver nitrate, zinc chloride, and tea tree oil, showing remarkable efficacy against all three bacterial strains. The observed percentages of antimicrobial activity are comparable to or higher than those reported in the literature for antimicrobial textiles targeting Gram-negative *E. coli* and *K. pneumonia*, as well as Gram-positive *MRSA* [51,59,60,61,62,63,64,65]. However, it is important to note that our results revealed a discrepancy in effectiveness, with HM4005 and HM4072 exhibiting high efficacy (>80% bacterial reduction) against the tested Gram-negative bacteria species but demonstrating minimal activity against Gram-positive *MRSA* (0% and 25% bacterial reduction for HM4005 and HM4072, respectively). Quaternary ammonium compounds (QACs), such as HM4005 and HM4072, are commonly used antiseptics to which some bacteria are known to be resistant, especially *MRSA* [37,66,67,68]. The prevalence and mechanisms of such resistance have not been fully uncovered yet; but it has been demonstrated that transcriptionally regulated Qac efflux pumps and membrane composition play a role [69]. Moreover, the variation in effectiveness could be attributed to the composition of commercial QAC formulations, which are typically dilute solutions containing a combination of multiple QACs. Recent studies exploring the structure–activity relationships of simple QAC scaffolds have suggested that incorporating multiple ammonium cations and reducing the structural rigidity of QACs may enhance their efficacy and therapeutic indices [70].

Antimicrobial textiles face a notable challenge regarding their durability and the potential risk of leaching. The longevity of an antimicrobial treatment is crucial for providing long-term protection. However, heat sterilization procedures can compromise the performance of antimicrobial agents, as the heat can cause molecular disintegration [28]. In healthcare settings, textiles undergo frequent sterilization, further affecting the antimicrobial coating’s durability. Our findings indicate that sterilization procedures have a significant impact on antimicrobial activity, particularly with QACs, which showed the lowest activity after sterilization. Nonetheless, silver nitrate and tea tree oil maintained their antimicrobial activity against Gram-negative bacteria (excluding *MRSA*) even after sterilization. Among the tested agents, zinc chloride-coated textiles exhibited the highest activity against all bacterial species, making zinc chloride the most durable antimicrobial agent.

Another important aspect to consider is whether new antimicrobial textiles are harmful to the environment and safe to use as PPE. These textiles, while effective against microbial infections, may release their antimicrobial agents over time through washing, abrasion, or prolonged use. This raises concerns about the potential negative effects on human health and the environment. Leaching of antimicrobial agents can contribute to bacterial resistance. Continuous release into the environment exposes bacteria to sublethal doses, promoting the growth of resistant strains. This undermines the long-term effectiveness of antimicrobial treatments and poses challenges in controlling infections [28]. Leached antimicrobial agents can enter water systems, harming aquatic organisms and disrupting ecosystems. Some agents are toxic to aquatic life, with far-reaching ecological implications when they accumulate in water bodies. To address this concern, it is important to develop antimicrobial textiles that minimize leaching. Out of the five tested antimicrobial agents, only the silver nitrate- and tea tree oil-treated textiles did not leach visibly, while apparent leaching was exhibited by the zinc chloride-, HM4005-, and HM4072-coated textiles. For these leaching agents, new strategies to covalently bond and polymerize these agents to the textile surface may be applied for more controlled or minimized leaching mechanisms. However, these might affect the antimicrobial efficacy of the compound, i.e., the most reactive part of a molecule within tea tree oil may also be what is used to bond with a textile.

Every day, millions of people around the world have symptoms of skin allergies, rashes, or itching, that can develop from chemicals used during the fiber manufacturing process, and from resins, dyes, or other chemicals used to treat textiles [71]. In more serious cases, chemicals can penetrate the deeper layers of skin causing systemic toxicity, leading to serious health concerns [72]. The reported in vitro cytotoxicity data of silver nitrate textiles are controversial [65,73]. When directly testing silver nitrate, in vitro cell culture studies have indicated toxic effects on immortal human skin keratinocytes, human erythrocytes, human neuroblastoma cells, human embryonic kidney cells, human liver cells, and human colon cells [74,75,76,77,78,79,80]. In contrast, silver nitrate-coated textiles, or the leachate of the textiles, did not cause toxic effects on human skin cells in vitro [65], aligned with our study’s findings. It has been shown that the toxic effects of silver nitrate nanoparticles are related to the release of free Ag^+^ in suspensions, which can be avoided with proper manufacturing and textile coating [65]. Ag^+^ release depends strongly on the textile’s nature, and the nature and concentration of the oxidant solution [65,73,81]. This unwanted release/leachate can be prevented during the textile manufacturing and antimicrobial coating process by using a technique that creates a strong bond between the textile fibers and silver nitrate. Zinc chloride nanoparticles act like a double-edged sword, with beneficial and harmful effects, i.e., they can effectively eliminate bacteria but also induce serious adverse effects [82,83]. It is well documented that zinc chloride causes skin irritations, gastrointestinal distress, diarrhea, nausea, and pulmonary issues. Accordingly, the European Commission restricts zinc chloride to a maximum 1% zinc concentration in preparations ready for use [84].

In recent years, the health safety of QACs has been a major topic of debate [35]. QACs have been associated with reduced fertility in mice, neural tube defects in rodents, mitochondrial dysfunction, and the inhibition of cholesterol biosynthesis [85]. In addition to these mechanistic in vivo studies on animals, QAC residue was identified in human blood to a total concentration of QACs of 10–150 nM [85]. Alarmingly, the levels of QACs in human blood (6.04 ng/mL) were significantly higher than those before the pandemic outbreak (3.41 ng/mL) due to the effect of COVID-19 and the increasing amount of disinfectants used [85]. Hydrophobic alkyl chains were reported to be strongly associated with the differential biodegradation and toxicity of QACs [86]. Previously, the cytotoxicity of tea tree oil used in aromatherapy has been reported [87]. The most common adverse effect was dermatitis, ranging from mild to severe, though its frequency and causality remain unknown [88]. To determine the biocompatibility of the tested textiles, we conducted cytotoxicity assays (resazurin and MTT) on HDF with leachate generated from antimicrobial-coated textiles. The data from cytotoxicity assays revealed that the leachates from silver nitrate- and tea tree oil-coated textiles were the least cytotoxic to HDFs compared to other test agents and can be considered the safest among the antimicrobial agents tested for preparing antimicrobial textiles. To our knowledge, this is the first study to carefully evaluate the cytotoxicity of tea tree oil-treated textile leachates on HDFs, showing that a low concentration of tea tree oil can be considered safe to human skin cells.

Lastly, the production techniques required for many antimicrobial finishes, including essential oils and metal nanoparticles, are prohibitively expensive (e.g., microencapsulation), thereby limiting large-scale production and commercial use [28]. It is worth noting that the process of applying antimicrobial agents to textiles can lead to an additional cost of 20–30% in textile manufacturing [27]. Taking all these into account, the antimicrobial treatment of textiles entails meeting various requirements to ensure their effectiveness, safety, durability, and environmental impact. These criteria include broad-spectrum efficacy against microorganisms, consumer safety, resilience during laundering, preservation of textile quality, compatibility with textile chemical processes, cost effectiveness, and absence of harm to manufacturers and the environment. To address these multiple criteria, a multicriteria decision-making technique, namely, the MULTIMOORA technique, was employed to select and rank the developed antimicrobial textile finishes. The MULTIMOORA is among the most practical MCDM methods and has been applied in various cases to solve complex decision-making problems, including personnel selection [89,90], risk assessment [91], project management [44], strategy assessment [92], ranking of renewable energy sources [93], textile supplier selection [94], and so on. Given the aforementioned applications of MULTIMOORA in previous research, this method is a completely effective method for evaluating and ranking antimicrobial textiles without subjective orientation in various phenomena [95]. This approach enables a comprehensive evaluation of quality characteristics and cost parameters in decision making. Through the application of multicriteria decision-making methods, conflicting criteria were systematically assessed, resulting in the identification of the optimal antimicrobial textile options. Overall, based on the results from these methods, silver nitrate was identified as the most appropriate choice, while HM4072 was considered the least suitable for the material selection problem.

This research highlights the effectiveness of applying multicriteria decision-making techniques to determine the optimal antimicrobial textile finish, providing a valuable tool in the fight against microbial infections. This knowledge can contribute to the development of more potent antimicrobial treatments, enabling enhanced protection for individuals in healthcare settings, public spaces, and other environments where the risk of infection is high. Furthermore, our research provides insights and guidelines for the textile industry to create antimicrobial textiles that meet stringent criteria for effectiveness, safety, and durability. By understanding the factors that influence antimicrobial activity, such as the composition of commercial formulations and the impact of sterilization procedures, textile manufacturers can make informed decisions in selecting appropriate antimicrobial agents and developing manufacturing processes that maximize the longevity and efficacy of these textiles. Moreover, the safety aspect of antimicrobial textiles cannot be overlooked. Our study evaluated the cytotoxicity of various antimicrobial agents and emphasizes the importance of selecting agents that are safe for human use. This knowledge enables industry to prioritize the development of textiles with minimal adverse effects on human health, ensuring the well-being of individuals who come into contact with these textiles on a regular basis.

## 5. Conclusions

In summary, our research was carried out to explore innovative methods to mitigate the health and economic implications of infections caused by the antibiotic-resistant ESKAPE pathogens. Specifically, we investigated the potential of antimicrobial textiles for PPE to disrupt pathogen transmission. Our study adopts a systematic approach, coupled with a mathematical decision-making methodology, to assess antimicrobial textiles that not only effectively target pathogens but also prioritize user health and cost effectiveness. Our research has significant implications for both the practical implementation of enhanced protection against microbial infections and the advancement of the textile industry. By leveraging the knowledge gained from our study, the industry can effectively develop antimicrobial textiles that not only combat infections but also prioritize safety and durability, catering to the diverse requirements of various environments and applications. This development will play a crucial role in improving public health, mitigating the spread of infectious diseases, and enhancing the overall well-being of individuals.

## Figures and Tables

**Table 1:** Calculated IC_50_ concentrations.

Target	Agent	IC_50_	SE	dfs
HDFs	Silver nitrate	0.004 mM	0.002	30
	Zinc chloride	1.621 mM	0.062	25
	HM4005	0.08%	0.076	28
	HM4072	0.001%	0.001	28
	Tea tree oil	0.15%	0.051	29

Abbreviations: degrees of freedom, dfs; standard error of IC_50_, SE; human dermal fibroblasts, HDFs.

**Table 2:** Summary of bacteriostatic reduction (BR) rate for each tested bacteria species.

Fabric Coating Agents Name	BR (%) *E. coli*	BR (%) *K. pneum*	BR (%) *MRSA*
Silver nitrate	99.87	100	84.05
Silver nitrate Sterilized	97.67	100	24.35
Zinc Chloride	99.87	100	99.71
Zinc Chloride Sterilized	99.85	100	97.83
HM4005	99.34	100	0
HM4005 Sterilized	65.78	0	36.03
HM4072	72.18	98.35	25.52
HM4072 Sterilized	0	21.48	0
Tea tree oil	100	100	99.13
Tea tree oil Sterilized	100	97.67	23.88

**Table 3:** Decision matrix for MCDM methods (rows represent the alternatives and columns show the criteria, with the first row indicating the criterion type).

	*AM* Properties *(% BR)*	*Toxicity (%* Viability*)*	*Heat Sensitivity (Binary)*	*Leaching (Binary)*	*Easy to* Use *(Binary)*	*Price* *USD*	*Odor (Binary)*	*Color (Binary)*
	BENEFIT	BENEFIT	COST	COST	BENEFIT	COST	COST	COST
***Silver Nitrate***	84.49	100	1	0	1	10.6	0	1
***Zinc chloride***	99.54	20	0	1	1	8.51	0	1
***HM4005***	50.19	40	1	1	0	2.2	0	0
***HM4072***	36.25	15	1	1	0	4.78	0	0
***Tea tree oil***	86.74	55	0	0	0	18.67	1	1

**Table 4:** Criteria weighting by experts (rows show the criteria and columns represent the experts’ weights, with the first row indicating the contribution of each expert’s input in the final ranking and the last column being the weighted average of all inputs).

	*Health Scientist/Engineer*			*Industry Partners*			*Final Weights*
***Expert ID***	**1**	**2**	**3**	**1**	**2**	**3**	
***Expert’s Opinion Weight***	**0.15**	**0.15**	**0.15**	**0.225**	**0.225**	**0.1**	**1**
***AM properties***	0.25	0.3	0.3	0.3	0.2	0.2	**0.2600**
***Toxicity***	0.25	0.3	0.3	0.2	0.2	0.25	**0.2425**
***Heat Sensitivity***	0.1	0.1	0	0.05	0.05	0.1	**0.0625**
***Leaching***	0.15	0.1	0.1	0.15	0.05	0.075	**0.1050**
***Easy Usage***	0.05	0.05	0	0.07	0.1	0.15	**0.0682**
***Price***	0.1	0.05	0.1	0.05	0.2	0.075	**0.1012**
***Odor***	0.05	0.05	0.1	0.08	0.1	0.1	**0.0805**
***Color***	0.05	0.05	0.1	0.1	0.1	0.05	**0.0800**
*SUM*	1	1	1	1	1	1	1

## Data Availability

The data generated in this study are available in the presented figures and tables.
